# Editorial: Targeting the endocannabinoidome in neurodegenerative disorders

**DOI:** 10.3389/fnagi.2022.1116635

**Published:** 2023-01-04

**Authors:** Hiroki Ishiguro

**Affiliations:** ^1^Department of Neuropsychiatry, Graduate School of Medicine, University of Yamanashi, Chuo, Japan; ^2^Department of Clinical Genetics, Graduate School of Medicine, University of Yamanashi, Chuo, Japan

**Keywords:** endocannabinoid, expanded endocannabinoid system, cannabinoid CB2 receptor, Alzheimer's Disease, Parkinson's Disease, N-Oleoyl-glycine, orexin

Current studies have unveiled the important roles of the endocannabinoid system (ECS) in multiple aspects of neural functions, including motor coordination, learning and memory, emotion and motivation, and psychiatric disorders such as mood disorders (de Melo Reis et al., 2021). ECS is composed of the two canonical receptor subtypes: type-1 cannabinoid (CB1R) and type 2 receptor (CB2R), as well as endocannabinoids (eCBs) and enzymes responsible for the synthesis and degradation of eCBs. Many studies have focused on each molecule of ECS using cannabinoid receptor ligands, such as CBD, to discover the role that ECS might play in psychiatric and neurodegenerative disorders. CB2R in particular became known as a major regulator of the activity of microglia, which is upregulated under inflammatory conditions (Komorowska-Müller et al., 2021). ECS could potentially be involved in those disorders by regulating monoamine systems (Mendiguren et al., 2021; Peters and Naneix, 2022) and GABAergic and cholinergic neurons.

In addition, expanded ECS, called “*endocannabinoidome*,” has been recognized to understand the molecular mechanism underlying various diseases and to develop novel therapeutics for them. The endocannabinoidome potentially includes hundreds of lipid mediators, more than 20 biosynthetic or inactivating enzymes, and more than 20 molecular targets, such as many previously identified nuclear receptors, ligand-activated ion channels, and orphan GPCRs (Veilleux et al., 2019; Morris et al., 2021). For example, N-Oleoyl-glycine is a lipid mediator that belongs to endocannabinoidome, which has recently gained attention for its protective effects in a mouse model of mild traumatic brain injury (Shahen-Zoabi et al., 2022). Besides, recent studies have also indicated an interaction between orexinergic and ECS, such as in addiction and fear (Deli et al., 2022; Ten-Blanco et al., 2022), while orexin's role in stress regulation and memory remains to be elucidated.

From a clinical perspective, depression varies in its clinical phenotypes at different onset ages. Our studies indicated the association of CB2R with patients with depression who had been diagnosed with major depressive disorders and had experienced a single episode of depression up until halfway through their lives but they did not include recurrent depression and bipolar disorders depression in the elderly (Onaivi et al., 2008), nor stress-related disorders in animal models (Ishiguro et al., 2018). ECS and related neural systems are needed to define the different phenotypes of depression. Since the early stages of Alzheimer's Disease (AD) and Parkinson's Disease (PD) are known to show a depressive phenotype, new perspectives regarding neuroinflammation and neuroprogression by endocannabinoidome could help unravel the molecular brain function underlying AD and PD.

While AD is characterized by an abnormal accumulation of β-amyloid (Aβ), eCBs have been shown to decrease Aβ-induced microglia activation and neuroinflammation (Vázquez et al., 2015; Schmöle et al., 2018). CB2R levels were significantly elevated in animal models of PD and postmortem studies of PD patients, and this increase correlated significantly with an increase in microglial activation, indicating the possible role of CB2Rs in PD (Concannon et al., 2015; Gómez-Gálvez et al., 2016).

This Research Topic focuses on neurodegenerative diseases and the comprehensive elucidation of endocannabinoidome in neurodegenerative disorders. It includes two original research projects: one brief research report and one review. In brief, each research piece indicates the role of CB2R in microglia and astroglia in AD-related cerebral amyloidosis as a suitable target for imaging neuro-inflammation and the role of the orexin system as endocannabinoidome in mild-to-moderate, age-related cognitive decline. Considering orexin's role as a stress regulator, it is also interesting to understand its potential role in the aforementioned different types of depression. A review summarizes the role of psychological and cellular stress on protein homeostasis *via* ECS, as a part of the neuro-immune system and the HPA axis, in degenerative conditions underlying AD. Another piece of original research shows that N-Oleoyl-glycine, which is known as a lipoamino acid that stimulates adipogenesis associated with the activation of CB1R, could have a promising therapeutic effect on PD. Hence, these carefully selected articles on this topic could point us toward understanding endocannabinoidome and the development of therapeutics for neurodegenerative disorders.

## Author contributions

The author confirms being the sole contributor of this work and has approved it for publication.
